# Pepper Mild Mottle Virus, a Plant Virus Associated with Specific Immune Responses, Fever, Abdominal Pains, and Pruritus in Humans

**DOI:** 10.1371/journal.pone.0010041

**Published:** 2010-04-06

**Authors:** Philippe Colson, Hervé Richet, Christelle Desnues, Fanny Balique, Valérie Moal, Jean-Jacques Grob, Philippe Berbis, Hervé Lecoq, Jean-Robert Harlé, Yvon Berland, Didier Raoult

**Affiliations:** 1 Unité de Recherche sur les Maladies Infectieuses et Tropicales Émergentes (URMITE), Centre National de la Recherche Scientifique (CNRS) Unité Mixte de Recherche (UMR) 6236 – Institut de Recherche pour le Développement (IRD) 3R198, Facultés de Médecine et de Pharmacie, Université de la Méditerranée, Marseille, France; 2 Pôle des Maladies Infectieuses et Tropicales Clinique et Biologique, Fédération de Bactériologie-Hygiène-Virologie, Centre Hospitalo-Universitaire Timone, Marseille, France; 3 Centre de Néphrologie et Transplantation Rénale, Centre Hospitalo-Universitaire Conception, Marseille, France; 4 Service de Dermatologie, Centre Hospitalo-Universitaire Sainte-Marguerite, Marseille, France; 5 Service de Dermatologie, Centre Hospitalo-Universitaire Nord, Marseille, France; 6 Institut National de la Recherche Agronomique (INRA), Unité de Recherche (UR) 407, Unité de Pathologie Végétale, Montfavet, France; 7 Service de Médecine Interne, Centre Hospitalo-Universitaire Conception, Marseille, France; Massachusetts General Hospital, United States of America

## Abstract

**Background:**

Recently, metagenomic studies have identified viable *Pepper mild mottle virus* (PMMoV), a plant virus, in the stool of healthy subjects. However, its source and role as pathogen have not been determined.

**Methods and Findings:**

21 commercialized food products containing peppers, 357 stool samples from 304 adults and 208 stool samples from 137 children were tested for PMMoV using real-time PCR, sequencing, and electron microscopy. Anti-PMMoV IgM antibody testing was concurrently performed. A case-control study tested the association of biological and clinical symptoms with the presence of PMMoV in the stool. Twelve (57%) food products were positive for PMMoV RNA sequencing. Stool samples from twenty-two (7.2%) adults and one child (0.7%) were positive for PMMoV by real-time PCR. Positive cases were significantly more likely to have been sampled in Dermatology Units (*p*<10^−6^), to be seropositive for anti-PMMoV IgM antibodies (*p* = 0.026) and to be patients who exhibited fever, abdominal pains, and pruritus (*p* = 0.045, 0.038 and 0.046, respectively).

**Conclusions:**

Our study identified a local source of PMMoV and linked the presence of PMMoV RNA in stool with a specific immune response and clinical symptoms. Although clinical symptoms may be imputable to another cofactor, including spicy food, our data suggest the possibility of a direct or indirect pathogenic role of plant viruses in humans.

## Introduction

Little is known regarding the viral flora present in the human gastrointestinal tract [1]–[5]. Recently, viral communities have been examined in non-diarrheic human stool in a systematic and unbiased fashion using metagenomics [6]–[7]. Interestingly, the vast majority of the recovered viral sequences corresponded to uncultured pathogenic plant RNA viruses [7]. The most abundant viral sequences isolated were from the *Pepper mild mottle virus* (PMMoV), with up to 10^9^ virions per gram of dry weight fecal matter. PMMoV is a non-enveloped, rod-shaped, single-stranded positive sense RNA virus classified in the genus *Tobamovirus*, which includes viruses extremely resistant to physical and chemical agents [8]–[9]. It is one of the major pathogens of *Capsicum* spp (chili peppers). Complementary data from Zhang *et al.*'s study have shown that PMMoV could be detected in non-diarrheic stool from 12 out of 18 individuals living in San Diego, USA or in Singapore, suggesting it might be geographically widespread, and in 3 out of 22 fresh and processed pepper samples. Moreover, the fecal PMMoV was viable and could infect host plants.

In the present study, we determined if PMMoV could be detected in commercial food products in our geographical area or the stool of patients and if these viruses were associated with biological and clinical symptoms.

## Materials and Methods

### Food products and human stool collection

Twenty-eight commercial food products were purchased in various grocery stores and supermarkets in Marseille (France) and stored at room temperature until processing (Table 1). They included four fresh peppers, thirteen manufactured products containing peppers, four products containing spice and seven products not known to contain pepper. For solid food products, 1 ml of the product was diluted in 1 ml of 1× PBS. Additionally, 565 stool samples were obtained between March and September 2008 for microbial diagnosis independent of the present study and approximately 1 ml collected randomly from each stool was stored in 1 ml of 1× PBS at +4°C. Of these samples, 357 were from adults (>18 years of age) and 208 were from children (<18 years of age). This study has been approved by our institutional ethics committee (Comité d'Ethique de l'IFR48, Université de la Méditerranée; review no. 09-001), that waived the need for consent.

**Table 1:** PMMoV RNA sequences recovered from food products.

no.	Presence of *Capsicum* spp or spice notified in food product composition	Type of food product	Food product	Origin	PMMoV RNA sequencing
1	No	Fruit or vegetable	Tomato	Unknown	Neg
2	No	Sauce	Salad sauce	Unknown	Neg
3	No	Sauce	Mayonnaise	France	Neg
4	No	Sauce	Oil	France	Neg
5	No	Sauce	“Exotic” sauce	France	Neg
6	No	Sauce	Tomato sauce	Unknown	Neg
7	No	Sauce	Vinegar	Unknown	Neg
8	Spice	Powder or dried food-product	Spicy powder no.1	Unknown	Pos
9	Spice	Powder or dried food-product	Spicy powder no.2	Mexico	Pos
10	Spice	Sauce	BBK sauce	Unknown	Pos
11	Spice	Sauce	Ketchup sauce	Europe	Neg
12	Yes	Fruit or vegetable	Green chili pepper	Morrocco	Neg
13	Yes	Fruit or vegetable	Red chili pepper no.1	Morrocco	Neg
14	Yes	Fruit or vegetable	Red chili pepper no.2	Unknown	Pos
15	Yes	Fruit or vegetable	Yellow chili pepper	Unknown	Neg
16	Yes	Powder or dried food-product	Cayenne pepper no.1	France	Pos
17	Yes	Powder or dried food-product	Cayenne pepper no.2	Unknown	Pos
18	Yes	Powder or dried food-product	Chili pepper and cumin powder	Mexico	Neg
19	Yes	Powder or dried food-product	Cumin powder	Unknown	Neg
20	Yes	Powder or dried food-product	Curry powder	Unknown	Pos
21	Yes	Powder or dried food-product	Red chili pepper powder	Unknown	Pos
22	Yes	Sauce	Chili pepper based-vinegar	Unknown	Neg
23	Yes	Sauce	Combava and chili pepper-based sauce	Unknown	Neg
24	Yes	Sauce	Harissa sauce	Unknown	Neg
25	Yes	Sauce	Tabasco sauce no.1	USA	Pos
26	Yes	Sauce	Tabasco sauce no.2	USA	Pos
27	Yes	Sauce	Tabasco sauce no.3	USA	Pos
28	Yes	Sauce	Tabasco sauce no.4	USA	Pos

### PMMoV RNA extraction

Viral RNA was extracted from 200 µl of the fecal supernatant or the solid food product suspensions following centrifugation at 10,000 *g* for 10 min or directly from the liquid food products by using the MagNA Pure LC RNA Isolation Kit (Roche Diagnostics, Meylan, France). The prepared RNA was directly analyzed or stored at −80°C until processing.

### Detection of PMMoV RNA by real-time PCR

PMMoV RNA was detected using a real-time reverse transcription (RT)-PCR assay with SuperScript III Platinum One-Step Quantitative RT-PCR System (Invitrogen Life Technologies, Carlsbad, Calif., USA) on an Mx3000P thermocycler (Stratagene, La Jolla, CA 92037 USA). Each 25 µl reaction contained 10 µl of the extracted viral RNA, 12.5 µl 2× reaction mix, 0.5 µl of SuperScript III/RT Platinum Taq mix, and 0.75 µl (10 pmol/µl) of each primer and 0.5 µl (10 pmol/µl) of probe described by Zhang *et al* [7]. Positive samples were further tested by RT-PCR amplification and sequencing, as well as electron microscopy.

### Infection of host plants with PMMoV RNA-positive food products

The viability and infectivity of PMMoV recovered from two PMMoV RNA-positive powder or dried food-products (no. 8, 17) and from a PMMoV RNA-positive Tabasco sample were tested by mechanical inoculation to host plants. Powder or dried food-products were crushed then mixed with an inoculation solution containing Na_2_HPO_4_ 0.03M and 0.2% (wt/vol) Na-diethyldithiocarbamate. Twenty ml of Tabasco sauce were mixed with 80 ml of 0.5 M phosphate buffer (Na_2_HPO_4_-KH_2_PO_4_, pH 7.2), then filtered, and 8% (vol/vol) of n-butanol was added. After agitation for 15 min, centrifugation was performed at 10,000 g for 30 min at 10°C, and the supernatant was collected and filtered. Then, 4% (wt/vol) Polyethyleneglycol 6000 and 4% (wt/vol) NaCl were added prior to stirring for 15 min then centrifugation at 10,000 g and 10°C for 15 min. Pellets were resuspended in 1 ml of 0.01 M phosphate buffer. Samples were mixed with 600 mesh Carborundum and 200 µl were inoculated mechanically to three leaves of two plants of *Nicotiana tabacum* var Xanthi NN, a hypersensitive host for PMMoV. Negative controls were the same numbers of plants either not inoculated or inoculated with the buffer only. All plants were kept for two weeks in the same unit of an insect-proof greenhouse. Local lesions typical of PMMoV infection were excised and crushed into 0.6 ml of inoculation buffer, and 350 µl of this solution were tested for the presence of PMMoV by RT-PCR using primers published by Hamada et al. [10], by electron microscopy, and by inoculation to one plant of *Capsicum annuum* var Yolo Wonder or *Nicotiana benthamiana*, two susceptible hosts for PMMoV.

### PMMoV RNA sequencing and phylogenetic analysis

PMMoV sequences corresponding to two different regions of the PMMoV genome were obtained by RT-PCR amplification using the SuperScript One-Step RT-PCR System (Invitrogen Life Technologies) and primers that had been designed with the SVARAP program [11]. The first targeted region is located near the 5′ end of the genome (primers used are PMMoV5pFwd: 5′-ATATCTGATGATGCAAGTTC-3′, 348 (the primer location was defined in reference to sequence GenBank accession nb NC_003630); PMMoV5p_Rev2: 5′-TAAACCTCTCTATTAGAGGC-3′, 928), and the second region corresponds to a fragment of the capsid gene (primers are PMMoVcaps_Fwd: 5′-CGTTAGGyAATCAGTTTCAA-3′, 5776; PMMoVcaps_Rev2: 5′-CGAACTAACTCATTCATGA-3′, 6070). For RT-PCR amplification, five µl of extracted viral RNA were added to 20 µl reaction solution containing 0.5 µl (10 pmol/µl) of primers, 12.5 µl of 2× reaction buffer, and 0.5 µl of RT/Platinum Taq mix. RT-PCR reaction was conducted under the following conditions: 45°C for 30 min for RT, 94°C for 2 min for initial denaturation, followed by 40 cycles of denaturation at 94°C for 30 s, annealing at 56°C for 45 s, elongation at 72°C for 2 min, then final elongation step at 72°C for 5 min. PCR products were purified with Sephadex G-50 Superfine on MAHVN 4550 plates (Millipore, Molsheim, France) then sequenced by using the amplification PCR primers and the Big Dye Terminator cycle sequencing kit version 1.1 on the ABI Prism 3130 genetic analyzer (Applied Biosystems, Branchburg, NJ, USA). The phylogenetic analysis was performed using Mega v.4.1 software (http://www.megasoftware.net/).

### PMMoV purification from Tabasco sauce

Tabasco sauce (50 ml) was centrifuged at low speed (2,000 rpm) for 20 min. The PMMoV-containing supernatant (30 ml) was then treated with 1 M NaCl (30 ml) at 4°C overnight and filtered using a 5 µm Millex-SV filter (Millipore). The filtrate was loaded onto a caesium chloride (CsCl) density gradient consisting of 1 ml layers of 1.7, 1.5 and 1.30 g/ml CsCl in 1× PBS. After ultracentrifugation (22,000 *g* for 2 h at 4°C), the 1.30–1.50 g/ml fraction containing the purified PMMoV (ρ = 1.31) was collected. This fraction was washed on a Microcon 100 centrifugal filter (Millipore) with 1× PBS.

### Electron microscopy study of PMMoV

A 40 µl aliquot of Tabasco sauce or fecal supernatant was deposited on a nickel 400 mesh Formvar carbon grid (EMS, Fort Washington, PA). After 15 min at 37°C, staining was performed with 1% ammonium molybdate for 10 sec. The stained grids were examined with a transmission electron microscope (Philips Morgagni 268D at 80Kv) after they were dried for 30 min at room temperature. The sensitivity of detection is estimated to be 10^6^–10^8^ virus particles/ml [12]. In addition, immunogold staining was performed, prior to electron microscopy, on PMMoV purified from PMMoV RNA-positive Tabasco sauce and stool, as follows: the electron microscopic grids were fixed with PBS and 1% glutaraldehyde for 2 min. After rinsing with PBS for 2 min, the grids were incubated twice for 5 min with 50 mM NH_4_Cl in PBS, preincubated twice for 2 min with Solution 1 (1% bovine serum albumine (BSA), 1% normal goat serum, 0.2% tween 20 in PBS) and incubated for 90 min with a polyclonal anti-PMMoV mouse antibody at 1∶1 dilution in Solution 1. These antibodies had been produced in two 6-week-old female Balb/C mice inoculated intraperitoneally with 1.3 µg of purified PMMoV using 400 µg aluminum hydroxide and 10 µg non-methylated oligonucleotides containing CpG motifs as an adjuvant, as previously described [13]. Blood collection was performed at day 38 following two booster doses administered at day 14 and day 28. After rinsing the stained grids with 0.1% BSA in PBS for 2 min, the grids were preincubated in Solution 2 (0.01% fish gelatin in PBS), transferred to a drop of gold-conjugated goat anti-mouse antibodies (diluted 1∶40 in Solution 2) for 60 min, washed six times with Solution 2 and twice with PBS for 10 min each. The grids were fixed in 2% glutaraldehyde for 15 min, then washed with PBS four times for 10 min and stained with 1% ammonium molybdate for 10 sec. Two negative controls were concurrently tested using antibodies derived from a non-immunized mouse or rotavirus instead of PMMoV.

### Anti-PMMoV ELISA

ELISA Nunc Maxisorp plates were coated with 100 µl PMMoV solution per well (0.6 µg/ml purified from a PMMoV RNA-positive Tabasco sauce) overnight at 4°C. All wells were machine-washed thrice with 300µl 0.1% Tween in PBS and blocked with 200 µl of PBS containing 5% dried skimmed milk for 30 min at room temperature with gentle agitation. The wells were washed with 0.1% Tween in PBS, and 100 µl of serum diluted in 3% milk /0.1% Tween in PBS was added. The plates were incubated for 1 h at room temperature and washed as described above. After washing, 100 µl of horseradish peroxidase–conjugated goat anti-human total Ig or IgM (Beckman Coulter, Marseille, France) diluted 1∶5000 in 3% milk /0.1%Tween in PBS was added. The plates were then incubated for 1 hr at room temperature and washed as described above. The plates were developed by adding 200 µl of ortho-phenylenediamine per well and the absorbance was read at 492 nm.

### Analysis of risk factors and biological and clinical signs associated with the presence of PMMoV RNA in stool or anti-PMMoV antibodies in serum

A case-control study was performed to assess the potential risk factors and the biological and clinical signs associated with the presence of PMMoV in the stool. Eighteen case-patients were compared to thirty-one control-patients with PMMoV RNA–negative stool. When possible, two control-patients from the same clinical units as the cases were selected for each case. The case- and control-patients were matched by age and sex. The variables analyzed included demographic characteristics, underlying diseases and clinical and biological signs. A Microsoft Excel spread sheet was used to enter the data. To identify risk factors for PMMoV detection, univariate analysis was performed with the Epi Info software version 3.3.2 (CDC, Atlanta, GA, USA). Proportions were compared by using the Yates Chi-square corrected test or the Fisher's exact test while continuous variables were compared by using either the ANOVA test or the Mann-Whitney/Wilcoxon two-sample test when the data were not normally distributed. For multivariate analysis, only variables with a *p* value ≤0.1 in univariate analysis were kept in the final model. Binary logistic regression analysis was performed using the SPSS software version 10 (Chicago, IL, USA).

## Results

### Detection of PMMoV RNA by real-time PCR

#### Food products

**Figure 1:**
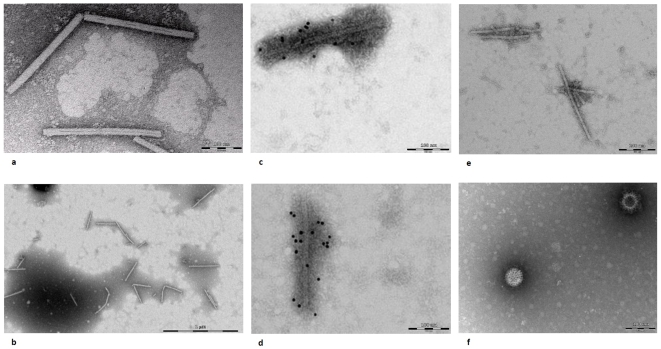
Electron microscopy of *Pepper mild mottle virus* (PMMoV)-RNA positive samples. Footnote: The samples analyzed by electron microscopy were (a) Tabasco sauce; (b) PMMoV purified from Tabasco sauce. The samples analyzed following immunogold staining were: (c) PMMoV purified from Tabasco sauce; (d) a PMMoV-RNA positive patient's stool sample; (e) PMMoV purified from Tabasco sauce stained with antibodies from a non immunized mouse (negative control no.1) and (f) a rotavirus-positive sample (negative control no.2).

PMMoV RNA sequences were recovered from twelve (57%) of the twenty-one pepper- or spice-containing food products (Table 1). Tabasco sauce contained the highest viral load, estimated to be nearly 10^7^ PMMoV RNA copies/ml based on a cycle threshold of 22 by real-time PCR or the presence of more than one viral particle/field by electron microscopy (Figure 1a).

#### Stool from children

PMMoV RNA was detected in the stool from only 1 child out of 137 (0.7%) using real-time PCR (Table 2). The presence of PMMoV RNA was confirmed by sequencing and viral particles compatible with PMMoV were observed using electron microscopy. The patient was a 5-year-old boy living in Marseille who was admitted to our institution because of abdominal pains, fever, asthenia and hyperventilation.

**Table 2:** Characteristics of individuals positive and negative for real-time PCR PMMoV RNA detection in stools.

	PMMoV RNA real-time PCR			*p*-values (Odds ratio (95% confidence limits for Odds ratio))
	Positive	Negative	All	
Children (<18 years of age)				
Nb of individuals	1	136	137	-
Nb of stools samples	1	207	208	-
Gender (sex ratio M∶F)	1∶0	1.08	1.09	-
Age (years; mean +/− SD (range))	5	2.9+/−4.1 (0–17.2)	2.9+/−4.1 (0–17.2)	-
Adults (>18 years of age)				
Nb of individuals (nb (%))	22 (7.2%)	282 (92.8%)	304	-
Nb of stools samples (nb (%))	23 (6.4%)	334 (93.6%)	357	-
Gender (sex ratio M∶F)	1.44	1.22		0.71
Age (years; mean +/− SD (range))	59.7+/−19.4 (19–85)	59.9+/−20.0 (18–96)		NS
Nb of patients with sequential stools samples (nb (%))	5 (22.7%^a^)	30 (10.6%^b^)	35	0.087 (2.47 (0.74–7.83))
Clinical units:				
Dermatology units (nb (%))	7 (35.0%)	13 (65.0%)	20	0.0000007 (9.66 (2.97–31.17))^c^
Nephrology units (nb (%))	4 (12.5%)	28 (87.5%)	32	0.074^d^
Internal Medicine units (nb (%))	3 (10.3%)	26 (89.7%)	29	0.12^e^
Other clinical units (nb (%))	8 (3.6%)	215 (96.4%)	223	-

Footnote: -, not tested; NS, not significantly statistically different; Nb, number. SD, standard deviation.

a % of individuals with PMMoV RNA-positive stools.

b % of individuals with PMMoV RNA-negative stools.

c Versus all other clinical units.

d Versus other clinical units than dermatology units.

e Versus other clinical units than dermatology units and nephrology units.

#### Stool from adults

The stool from 22 of 304 (7.2%) adults was positive for PMMoV by real-time PCR (Table 2). This proportion was significantly higher than the incidence in children (*p* = 0.0043). PMMoV RNA sequence could be recovered from all but two patients, whereas PMMoV-like viral particles were observed, by electron microscopy, in the stool from all but one patient, who was found positive for PMMoV RNA sequencing (Figure 1d) . The proportion of PMMoV RNA–positive patients was significantly higher in Dermatology Units than in other clinical units (7/20 (35.0%) versus 15/284 (5.3%); *p* = 7×10^−7^) (Table 2). Clinical and biological data were obtained from 18 of the 22 PMMoV-positive patients and compared to the characteristics of 31 patients with PMMoV-negative stool. By univariate analysis, abdominal pains, diverticulosis or diverticulitis, and fever were significantly associated with PMMoV RNA-positive stool (Table 3). No biological sign was significantly associated with the presence of PMMoV RNA. By multivariate analysis, using a model including age, gender and the variables significantly associated with the case-patients in the univariate analysis, fever (*p* = 0.0447 (relative risk (RR), 5.290 (95% confidence limits for RR (CI95), 1.040–26.911)), abdominal pains (*p* = 0.0376 (7.069 (1.119–44.660)), and pruritus (*p* = 0.0460 (6.131 (1.033–36.402)) were independently associated with the presence of PMMoV RNA. In addition, an interaction was observed between abdominal pains and diverticulitis. Overall, the model fitted the data well (Hosmer-Lemeshow test equal to 0.94). Three of four patients with concurrent abdominal pains and fever and the two patients who had abdominal pains and pruritus were PMMoV RNA-positive.

**Table 3:** Univariate analysis for risk factors and biological and clinical features in association with the detection by real-time PCR of PMMoV RNA from stools.

	PMMoV RNA real-time PCR (Nb of patients (%))			P-values (Odds ratio (95% confidence limits for Odds ratio))
	Positive (n = 18)	Negative (n = 31)	All (n = 49)	
Age>63 years^a^	9 (50)	17 (55)	26 (53)	0.458
Male gender	12 (67)	18 (58)	30 (61)	0.286
Stools sample collected in:				
Dermatology units	7 (39)	9 (29)	16 (33)	0.249
Nephrology units	4 (22)	8 (26)	12 (25)	0.532
Infectious diseases units	2 (11)	4 (13)	6 (12)	0.616
Internal medicine units	1 (6)	2 (7)	3 (6)	0.698
Endocrinology units	1 (6)	2 (7)	3 (6)	0.698
Hepato-gastro-enterology units	1 (6)	2 (7)	3 (6)	0.698
Cardiology units	1 (6)	2 (7)	3 (6)	0.698
Neurological surgery units	1 (6)	2 (7)	3 (6)	0.698
Any among the following source of immune suppression	7 (39)	10 (32)	17 (35)	0.325
HIV infection/AIDS	0 (0)	1 (3)	1 (2)	0.633
Solid organ transplantation	2 (11)	2 (7)	4 (8)	0.470
Kidney transplantation	2 (11)	1 (3)	3 (6)	0.302
Hematopoietic stem cell transplantation	0 (0)	1 (3)	1 (2)	0.633
Any immunosuppressive therapy	6 (33)	9 (29)	15 (31)	0.379
Corticotherapy	4 (22)	6 (19)	10 (20)	0.542
**Clinical signs:**				
Any among the following clinical signs	13 (72)	17 (55)	30 (61)	0.124
Nausea and/or vomiting	1 (6)	1 (3)	2 (4)	0.605
Diarrheoae	5 (28)	10 (32)	15 (31)	0.381
**Abdominal pains**	**7 (39)**	**2 (7)**	**9 (18)**	**0.0079 (2.83 (1.53–5.22))**
Oesophagitis	2 (11)	2 (7)	4 (8)	0.470
Gastritis	0 (0)	1 (3)	1 (2)	0.633
Gastric or duodenal ulcer	1 (6)	1 (3)	2 (4)	0.605
Colitis	0 (0)	2 (7)	2 (4)	0.395
Proctitis	1 (6)	0 (0)	1 (2)	0.367
Polyp	1 (6)	0 (0)	1 (2)	0.367
Prostatitis	1 (6)	1 (3)	2 (4)	0.605
Constipation	1 (6)	0 (0)	1 (2)	0.367
**Any among the two following symptoms**	**3 (17)**	**0 (0)**	**3 (6)**	**0.044 (3.07 (2.02–4.65))**
Diverticulosis	1 (6)	0 (0)	1 (2)	0.367
Diverticulitis	2 (11)	0 (0)	2 (4)	0.130 (2.94 (1.97–4.37))
Blood in stools	1 (6)	1 (3)	2 (4)	0.605

	PMMoV RNA real-time PCR (Nb of patients (%))			P-values (Odds ratio (95% confidence limits for Odds ratio))
	Positive (n = 18)	Negative (n = 31)	All (n = 49)	
**Clinical signs ** ***(continuation)*:**				
Any among the following clinical signs	9 (50)	8 (26)	17 (35)	0.051 (1.88 (0.92–3.84))
Pruritus	5 (28)	3 (10)	8 (16)	0.107 (1.97 (0.98–3.97))
Urticaria	1 (6)	0 (0)	1(2)	0.367
Eczema	2 (11)	1 (3)	3 (6)	0.302
Skin rash	0 (0)	2 (7)	2 (4)	0.395
Bullous skin rash	0 (0)	1 (3)	1 (2)	0.633
Dermatosis	0 (0)	1 (3)	1 (2)	0.633
Dermo-hypodermiitis	1 (6)	0 (0)	2 (4)	0.367
Erysipeloid rash	1 (6)	1 (3)	2 (4)	0.605
Erythrodermia	0 (0)	0 (0)	0 (0)	-
Psoriasis	0 (0)	1 (3)	1 (2)	0.633
Purpura	1 (6)	1 (3)	2 (4)	0.605
Furonculosis	1 (6)	0 (0)	1 (2)	0.367
Impetigo	1 (6)	0 (0)	1 (2)	0.367
Vitiligo	0 (0)	1 (3)	1 (2)	0.633
Hepatomegaly	2 (11)	2 (7)	4 (8)	0.470
Cirrhosis	1 (6)	2 (7)	3 (6)	0.698
**Fever**	**7 (39)**	**4 (13)**	**11(22)**	**0.042 (2.20 (1.13–4.30))**
Other clinical signs:				
Anorexia	0 (0)	3 (9)	3 (6)	0.244
Asthenia	3 (17)	6 (19)	9 (18)	0.567
Renal insufficiency	3 (17)	14 (45)	17 (35)	0.025 (0.38 (0.13–1.12))
Dialysed renal insufficiency	3 (17)	4 (13)	7 (14)	0.512
Diabetes mellitus	5 (28)	5 (16)	10 (20)	0.268
Vascularitis	0 (0)	0 (0)	0 (0)	-
Increased blood pressure	9 (50)	14 (45)	23 (46)	0.377
Joint pains	2 (11)	0 (0)	2 (4)	0.130 (2.94 (1.97–4.37))
Asthma	1 (6)	1 (3)	2 (4)	0.605
Allergy	2 (11)	1 (3)	3 (6)	0.302
Thrombosis history	2 (11)	2 (7)	4 (8)	0.470
Pneumonia	5 (28)	3 (10)	8 (16)	0.107 (1.97 (0.98–3.97))
Lung clinical symptoms	5 (28)	5 (16)	10 (20)	0.268

	PMMoV RNA real-time PCR (Nb of patients (%))			P-values (Odds ratio (95% confidence limits for Odds ratio))
	Positive	Negative	All	
**Biological signs:**				
Increased levels of blood markers of inflammation^b^	9 (64)	17 (61)	26 (62)	0.419
Leukocyte count >10.000/mm^3^	2 (11)	9 (31)	11 (23)	0.111
Lymphopenia	9 (56)	12 (44)	23 (50)	0.238
Polynuclear neutrophil count >10,000/mm^3^	3 (18)	8 (28)	11 (24)	0.349
Polynuclear eosinophil count >500/mm^3^	2 (11)	2 (7)	4 (8)	0.484
Increased levels of IgE antibodies	2 (11)	4 (13)	6 (12)	0.616
Antinuclear antibodies positivity	1 (6)	0 (0)	1 (2)	0.367
Increased levels of liver enzymes	6 (33)	9 (30)	15 (31)	0.406
ALT >45 IU/L	0 (0)	4 (24)	4 (16)	0.188
Gammaglutamyltransferase >45 IU/L	3 (38)	5 (29)	8 (32)	0.513
Bilirubinemia >35 µmol/L	1 (13)	0 (0)	1 (5)	0.381
**Positive electron microscopy**	**17 (94)**	**0 (0)**	**17 (53)**	**<10^−6^ (15.00 (2.26–99.64))**
Total anti-PMMoV antibodies positivity in serum	6 (75)	2 (29)	8 (53)	0.100 (2.63 (0.76–9.05))
**IgM Anti-PMMoV antibodies positivity in serum**	**8 (100)**	**3 (43)**	**11 (73)**	**0.026 (nc)**

Footnote: -. undefined; Nb, Number; Nc, not calculable; *p* values <0.05 are in boldface.

a 63 years was the median age of the adult patients tested in the case-control study.

b Blood markers of inflammation were elevated erythrocyte sedimentation rate, and/or C-reactive protein and fibrinogen levels in serum.

### Infection of host plants with PMMoV RNA-positive food products

**Figure 2:**
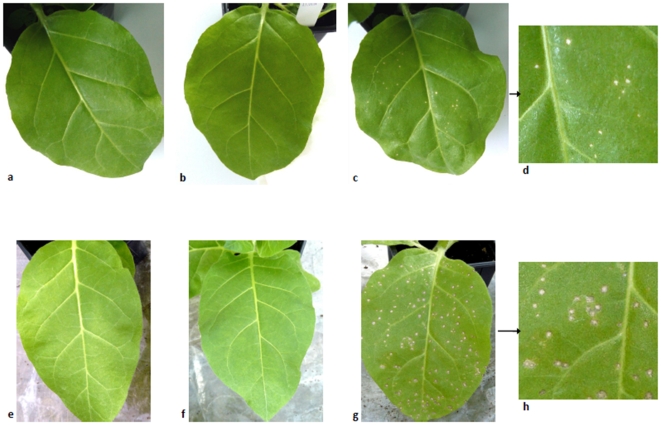
Local lesions typical of PMMoV infection on *Nicotiana tabacum* cultivar Xanthi NN plants following inoculation of processed PMMoV RNA-positive food products. Footnote: Leaves of *Nicotiana tabacum* cultivar Xanthi NN plants (a, e) non inoculated (negative control); (b, f) inoculated with buffer only (negative control); (c, and detail: d) inoculated with a processed PMMoV RNA-positive Tabasco sample; (g, and detail: h) inoculated with a PMMoV RNA-positive food product (no. 17; Table 1).

All *N. tabacum* cultivar Xanthi NN plants inoculated with each of the three PMMoV RNA-positive food products developed local lesions typical of PMMoV infection within 5–7 days post-inoculation (Figures 2a–h). These lesions were observed on the three inoculated leaves for each plant, whereas control plants remained devoid of symptoms during an observation period of two weeks post-inoculation. PMMoV RNA was detected using RT-PCR from local lesions, PMMoV-like viral particles were observed by electron microscopy, and systemic mosaic symptoms typical of PMMoV were observed within 2–3 weeks post-inoculation on *C. annuum* cultivar Yolo Wonder or *N. benthamiana*.

### PMMoV RNA sequencing and phylogenetic analysis

**Figure 3:**
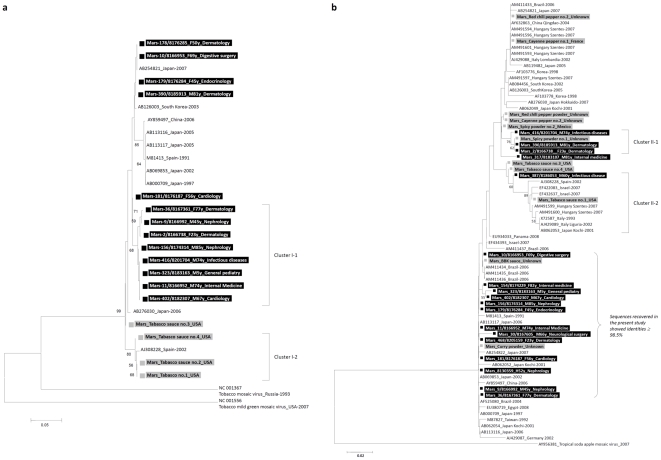
Phylogenetic comparison of PMMoV nucleotide sequences recovered in the present study and corresponding (a) to a region located near the 5′ end of the genome or (b) to a region in the capsid gene to PMMoV sequences selected from GenBank. Footnote: The phylogenetic trees were constructed using the neighbor-joining method based on the 5′ region (a) of the PMMoV genome or a region in the capsid gene (b). The PMMoV sequences recovered from patients' stool in the present study are in the boldface, white font and are indicated by a black square. Their name is labeled as follows: Mars_Laboratory identification number_Gender-Age_Clinical unit where the patient received care. The PMMoV sequences recovered from food products in the present study are in a boldface and are indicated by a gray square. Their name is labeled as follows: Mars_Name of the food product_Country where food product was manufactured. The remaining PMMoV sequences were obtained from GenBank. Their name is labeled as follows: GenBank Accession no._Coutry of origin_Year of submission to GenBank. Bootstrap values are indicated when greater than 50% as a percentage obtained from 1,000 resamplings of the data. The scale bar indicates the number of nucleotide substitutions per site. The sequence of *Tobacco mild green mosaic virus* (GenBank Accession No. NC_001556) and *Tropical soda apple mosaic virus* (GenBank Accession No. AY956381) were used as outgroups.

PMMoV sequences corresponding to the 5′ region of the PMMoV genome were obtained from four Tabasco sauce samples and thirteen patients, whereas sequences corresponding to a fragment of the capsid protein gene were obtained from eleven food products, including three Tabasco sauce samples, and eighteen patients (Figures 3a and 3b, respectively; GenBank accession numbers GQ427021–GQ427066). The BLAST analysis of the nucleotide sequences from the 5′ region produced only ten relevant hits, after which the sequences no longer corresponded to PMMoV (http://blast.ncbi.nlm.nih.gov/Blast.cgi). The sequences recovered from Tabasco sauce tended to cluster separately from those recovered from patients (Figure 3a). The mean nucleotide identity was 96.5±2.2% (range, 90.9–100%) for all PMMoV sequences, 96.4±2.4% (91.0–100%) for sequences recovered in the present study, 97.8±1.9% (93.9–100%) for sequences obtained from GenBank and 67.1±1.0% (64.1–68.6) with a *Tobacco mosaic virus* sequence. For the sequences of the capsid protein gene, the mean nucleotide identity was 97.1±1.8% (90.8–100%) for all PMMoV sequences, 97.2±1.9% (91.3–100%) for sequences recovered in the present study, 97.5±2.4% (90.8–100%) for sequences obtained from GenBank and 76.2±0.9% (73.4–77.7%) with *Tropical soda apple mosaic virus* sequence. One cluster (cluster II-1) contained four sequences from the present study (Figure 3b), and 100% nucleotide identity was observed between the sequences from two patients that received care in a Dermatology Unit and from a spicy powder.

### Anti-PMMoV Total Ig and IgM ELISA

**Figure 4:**
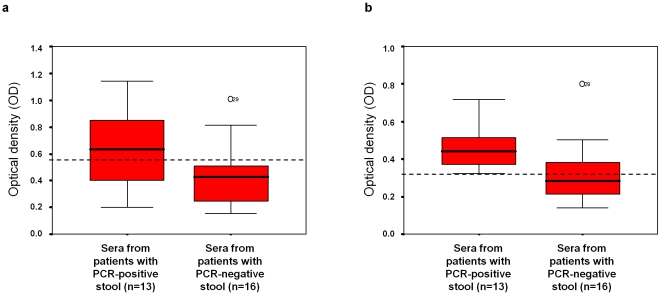
Comparison of the serum anti-PMMoV antibody level (a, total antibody; b, IgM) between individuals with PMMoV RNA-positive and PMMoV RNA-negative stool. The dashed lines indicate the optical density cut-off values that were chosen for positive PMMoV serology using the receiver operating characteristic (ROC) curve test.

The level of serum anti-PMMoV total and IgM antibodies was examined by ELISA in a subset of 29 adult patients, 13 of which had PMMoV-positive stool, as detected by PCR (Figures 4a and 4b). For the analysis of the IgM anti-PMMoV level, the optical density (OD) for the substrate and antibody controls was 0.079 and 0.099, respectively. The mean OD was significantly higher in patients with PMMoV RNA than in the controls (0.472 versus 0.325, *p* = 0.013 (ANOVA); Figure 4b). Using the receiver operating characteristic (ROC) curve test with a sensitivity of 100% and a specificity of 62%, a value of 0.320 was considered positive for the detection of PMMoV-specific IgM (AUC = 0.808; 95% confidence interval: 0.645–0.971; *p* = 0.005). The proportion of serum samples with an OD greater than 0.320 was significantly higher for patients with PMMoV RNA-positive stool than for the controls (*p* = 0.0004). With this threshold, all 13 serum from patients with PCR PMMoV-positive stool were seropositive, whereas 10/16 (63%) sera from patients with PCR PMMoV-negative stool were seronegative. Thus, the positive and negative predictive values for having PCR-positive stool were 68% and 100%, respectively. For the analysis of the total anti-PMMoV level, the optical density (OD) for the substrate and antibody controls was 0.051 and 0.071, respectively. The mean OD was significantly higher in patients with PMMoV RNA than in the controls (0.663 versus 0.444, *p* = 0.028; Figure 4a). A cut-off value of 0.565 (estimated using the ROC curve test with a sensitivity of 69% and a specificity of 81%) was used (AUC = 0.724; 95% confidence interval: 0.529–0.918; *p* = 0.041). The proportion of serum samples for which the optical density (OD) was greater than 0.565 was significantly higher for patients with PMMoV-positive stool than for those with PMMoV-negative stool (*p* = 0.0070). Using this threshold, 9 (69%) of the 13 sera from patients with PCR PMMoV-positive stool were seropositive, whereas 13 (82%) of the 16 sera from patients with PCR PMMoV-negative stool were seronegative. Additionally, serological patterns indicating past infection (i.e., a positive total antibody result, but a negative IgM result) and negative total antibody and IgM testing were observed in two and eight PMMoV-negative patients, respectively, but in none of the PMMoV-positive patients (*p* = 0.0004). Finally, in the case-control study (Table 3), positive testing for anti-PMMoV IgM antibody was significantly more frequent in case-patients than in control-patients.

## Discussion

We detected PMMoV RNA by real-time PCR in stool from 22 of 304 (7.2%) adult patients and 1 of 208 children who received care in public hospitals. The present study therefore confirms the recent finding by Zhang *et al.* who identified PMMoV as the major RNA virus in human stool in a metagenomic study [7]. However, we found a much lower prevalence of PMMoV RNA-positive stool by real-time PCR among adult patients than Zhang *et al.*, who detected the viral genome in 12 of 18 fecal samples (66.7%) using RT-PCR. This difference might be explained either by a lower sensitivity for real-time PCR or the lower exposure to PMMoV in our patients. We recovered PMMoV RNA sequences from 57% of food products containing pepper or spice and purchased from grocery stores in Marseille (southeastern France), indicating that these products represent a common source of viruses through ingestion. In addition, in food products and patient's stool, we could visualize by electron microscopy complete viruses whose shape and size are consistent with PMMoV and that reacted with PMMoV-specific antibodies in the immunogold staining experiments. Moreover, 46 new PMMoV sequences corresponding to two regions of the viral genome were recovered from food products or patients' stool. Four phylogenetic clusters were identified, and one of them (II-1) included sequences from both patients and a putative food source. Furthermore, three PMMoV RNA-positive food products were found to contain virus particles infectious to host plants.

We also identified statistically significant differences in the occurrence of fever, abdominal pains, and pruritus and the detection of specific immune responses to PMMoV in the case-control study. We, therefore, believe that we provide the first evidence that plant viruses may cause disease in humans. Fever and abdominal pains, which were significantly more common in PMMoV-positive patients (39% for both clinical features) than in patients with PMMoV-negative stool (13% and 7%, respectively), were also present in the only PMMoV RNA-positive child. These symptoms might not be caused by PMMoV but by consumption of spicy food. Otherwise, they may be fortuitously related to an associated unknown infectious agent or to an allergic or toxic cofactor. Nevertheless, the presence of a specific immune response to PMMoV reinforces the hypothesis that this plant virus was the cause of the symptoms in these patients. Indeed, anti-PMMoV IgM antibodies were detected in all PMMoV-positive patients, significantly more frequently than in the controls. The concurrent detection of PMMoV RNA and antibodies to PMMoV indicates that this virus is not a neutral component of the human gut flora. On the contrary, our findings suggest that PMMoV may infect humans and cause clinical symptoms. We believe that these preliminary data may prompt further studies on the pathogenic role of PMMoV in humans. Finally, the higher prevalence of PMMoV RNA among patients who received care in Dermatology Units deserves further attention, as it was high (35%).

To date, plant viruses and vertebrate viruses are believed to exist in two distinct and non overlapping biological niches, and they are not known to share common hosts in the plant or animal kingdoms [14]–[15]. Therefore, to date, plant viruses have not been described as pathogens for vertebrates or humans or known even to infect them [16]. This paradigm is underscored by the growing body of data about the use of plant virus–based vaccines, which are considered as safe tools that might be useful in treating chronic viral infections and cancer in the future [17]–[20]. However, the boundaries between plants and animals might not be so hermetical for plant viruses, and, to our knowledge, no experimental model has been described that would allow us to rule out a pathogenic role for plant viruses in vertebrates. The recent work of Zhang et al. challenged this dogma, as it was neutral, technology-driven research that was not trying to confirm a former hypothesis [7], [21]. That work identified plant viruses as the most common viral inhabitants of the human gut flora, PMMoV being the most prevalent. In addition, a recent study demonstrated that PMMoV is widespread in wastewater from the United States of America (USA), at concentrations ranging between 8.0×10^5^ and 2.2×10^7^ copies/ml [22]. Beyond these findings, our results, together with previous data, suggest that the relationship between plant viruses and animals may deserve further re-evaluation using modern microbiologic tools (Table 4). The oral administration of *Cowpea severe mosaic virus*, another plant virus, has been previously shown to induce a durable and systemic immune response in mice without requiring the co-administration of an adjuvant [23], and similar results have been observed in mice using *Alfalfa mosaic virus* and chimeric plant virus particles [24]–[25]. In addition, some plant viruses can replicate in insect tissues, as illustrated by *Tomato spotted wilt virus* and *Maize rayado fino virus* [26]–[28]. Furthermore, a reduction in the lifespan and fecundity of insects (*Bemisia tabaci*) during infection with *Tomato yellow leaf curl virus* has been previously observed, indicating that this plant virus had some features of an insect pathogen [29]. It has been also suggested, based on viral sequence analysis, that during evolution, plant-infecting viruses (previously known as plant circoviruses and recently reclassified in the genus *Nanovirus*) switched hosts to infect a vertebrate and then recombined with a vertebrate-infecting virus [16]. Moreover, it has been shown recently that *Tomato spotted wilt virus* could replicate in two human cell lines, HeLa and diploid fibroblasts [30]. This virus is one of the most important plant pathogens worlwide, and it causes mild infection on its main insect vector, *Frankliniella occidentalis*, in which it induces a strong immune response [26]. Finally, the possible involvement of plant viruses in human diseases was questioned several decades ago. Indeed, culturable *Tobacco mosaic virus* could be recovered from sputum and thoracentesis fluids obtained from cigarette smokers with a history of pulmonary disease, including lung cancer [31]–[33]. Taken together, these experimental data, coupled with the previous results reported by Zhang *et al.* and our findings, favor the hypothesis of a pathogenic role for PMMoV. It should be highlighted that PMMoV, like other tobamoviruses, are known to be extraordinarily resistant to heat and desiccation [34]–[35]. Moreover, although this deserves to be investigated in further studies, the viability and infectivity to host plants were evidenced for PMMoV recovered from food products in the present study and from stools in Zhang et al.'s study [7]. Additionally, we visualized complete viral particles in stool by electron microscopy.

**Table 4:** Summary of postulates and findings about interactions of PMMoV with the host immune system, its replication in non-plant tissues, and its potential link with clinical signs.

Postulate	Plant virus	Finding	Reference
**PMMoV may interact with the host immune system**	*Pepper mild mottle virus*	Specific immune response as indicated by positive anti-PMMoV IgM antibody testing in patients with PMMoV in stool	Present study
	*Cowpea severe mosaic virus*, *Alfalfa mosaic virus*	Evidence of systemic immune response (specific synthesis of IgG and IgA in sera) in mice immunized orally with *Cowpea severe mosaic virus* (CPSMV) in the absence of immunoadjuvants. Similar results were observed with viral particles of *Alfalfa mosaic virus* and chimeric plant virus particles	[23]–[25]
	*Tomato spotted wilt virus*	Induction of a strong immune response in *Frankliniella occidentalis*, its main insect vector	[26]
	*Pepper mild mottle virus*	High PMMoV loads in stool of patients and food products containing pepper	[7], Present study
**PMMoV may replicate in humans**	*Pepper mild mottle virus*	High PMMoV loads in stool of patients and food products containing pepper	[7], Present study
	*Pepper mild mottle virus*, *Tobamovirus*	High viral stability	[8], [34], [35]
	*Pepper mild mottle virus*	PMMoV detected from human stool is viable	[7]
	*Tomato spotted wilt virus*; *Maize rayado fino virus*	Some plant viruses are able to multiply in non-plant tissue (in insect tissues)	[26]–[28]
	*Nanovirus*	Evidence that a plant virus switched hosts to infect a vertebrate	[16]
	*Tomato spotted wilt virus*	Expression of a viral polymerase-bound factor turned human cell lines, HeLa and diploid fibroblasts, permissive to *Tomato spotted wilt virus*	[30]
	*Tobacco mosaic virus*	Plant virus particles could be assembled in *Escherichia coli*	[36]
**PMMoV may induce clinical signs**	*Tomato yellow leaf curl virus*	Reduction of lifespan and fecundity of the insect vector (*Bemisia tabaci*) during viral infection	[29]
	*Tobacco mosaic virus*	Recovery of culturable tobacco mosaic virus from sputum and thoracentesis fluids obtained from cigarette smokers with a history of pulmonary disease	[31]–[33]
	*Pepper mild mottle virus*	Fever and abdominal pains are significantly more frequent in patients with PMMoV-positive stool	Present study

In summary, we confirmed that humans may carry a high PMMoV load, likely acquired from food products, and we documented that PMMoV might not only be a common inhabitant of the human gut but may also interact with the human immune system and cause clinical symptoms. These results should prompt further studies to re-evaluate whether or not plant viruses, including PMMoV, may have a pathogenic role in humans.
