# Correction: Pepper Mild Mottle Virus, a Plant Virus Associated with Specific Immune Responses, Fever, Abdominal Pains, and Pruritus in Humans

**DOI:** 10.1371/journal.pone.0347179

**Published:** 2026-04-14

**Authors:** The PLOS One Editors

The *PLOS One* Editors issue this notice to update the previously published Expression of Concern on this article [1,2].

This study reports the use of stool samples collected from human participants, including minors, between March and September 2008. The article reports approval from the Institut Fédératif de Recherche (IFR) 48 (review no. 09−001), Marseilles, France. The Expression of Concern [2] was issued due to concerns about several approvals issued by this institution and concerns that the article did not report approval from a Comité de Protection des Personnes (CPP).

A representative of the Aix-Marseille Université Ethics Committee stated that the institutional investigation into the ethics concerns concluded this article meets ethical standards. They commented that the study did not involve invasive or risky sampling as the study is based solely on the analysis of stool samples collected as part of routine patient care, and that the study did not require ethics approval from a Comité de Protection des Personnes according to French law. The representative provided the ethics approval document #09−001 for editorial review.

Ethics approval document #09−001 was issued by the Comité d’Ethique de l’IRF48 on January 16, 2009, for a study titled “*Recherche de virus des piments dans des selles adressées pour examen microbiologique*”. The document specifies approval for an epidemiological study using previously collected clinical stool samples obtained for microbiological analysis, and the study described in the document appears to match the study reported in this article.

Based on the information and documentation received, the original ethics approval concerns are resolved. However, in investigating this matter PLOS identified potential competing interests between the IFR 48 ethics committee that granted the ethics approval and one or more of the article's authors.

Additionally, concerns were raised about similarities between Fig 2a and Fig 2b. The authors did not respond to editorial communications, but an individual identifying themselves as co-author HL commented on Pubpeer [3] that these panels inadvertently present the same leaf and stated that the data underlying these results are no longer available. Although this concern remains unresolved, Fig 2e and Fig 2f present a second set of controls representing the same conditions, and so the results remain supported.

This Correction supersedes the prior Expression of Concern [2].
